# Fifty-Two-Week Results of Clinical and Imaging Assessments of a Patient with Rheumatoid Arthritis Complicated by Systemic Sclerosis with Interstitial Pneumonia and Type 1 Diabetes despite Multiple Disease-Modifying Antirheumatic Drug Therapy That Was Successfully Treated with Baricitinib: A Novel Case Report

**DOI:** 10.1155/2019/5293981

**Published:** 2019-07-09

**Authors:** Yuya Fujita, Masao Nawata, Atsushi Nagayasu, Kazuki Someya, Kazuyoshi Saito, Yoshiya Tanaka

**Affiliations:** ^1^The Department of Rheumatology, Tobata General Hospital, The First Department of Internal Medicine, School of Medicine, University of Occupational and Environmental Health, Kitakyushu, Japan; ^2^The First Department of Internal Medicine, School of Medicine, University of Occupational and Environmental Health, Kitakyushu, Japan

## Abstract

Baricitinib is a Janus kinase 1/2 (JAK1/2) inhibitor used in the treatment of rheumatoid arthritis. A 71-year-old woman with rheumatoid arthritis complicated by systemic sclerosis and type 1 diabetes that were resistant to multiple disease-modifying antirheumatic drugs started treatment with baricitinib. After baricitinib administration, the disease activity of her rheumatoid arthritis was attenuated from the early stage of treatment, and the effect was maintained for up to 52 weeks. In addition, the skin sclerosis in systemic sclerosis showed an improvement. Regarding the influence on type 1 diabetes, the required daily dose of insulin and hemoglobin A1c (HbA1c) levels decreased. To date, no studies have demonstrated the effectiveness of baricitinib on systemic sclerosis or type 1 diabetes. We report that baricitinib was effective for systemic sclerosis and type 1 diabetes, as well as for rheumatoid arthritis, for up to 52 weeks.

## 1. Introduction

In recent years, the clinical efficacy of targeted synthetic disease-modifying antirheumatic drugs (tsDMARDs) that selectively inhibit Janus kinase (JAK), which is critical for lymphocyte signaling, has been demonstrated in patients with rheumatoid arthritis [1, 2]. According to the European League Against Rheumatism (EULAR) recommendations published in 2016, JAK inhibitors were recognized as equivalent to biological DMARDs [3, 4]. Among JAK inhibitors, baricitinib is expected to be effective for controlling various immune responses by selectively inhibiting JAK1/2 [5–7]. Herein, we present a case of rheumatoid arthritis complicated by systemic sclerosis associated with interstitial pneumonia and type 1 diabetes in a patient who was resistant to multiple DMARDs but was successfully treated with baricitinib for up to 52 weeks and discuss the analysis of the joint ultrasonography images of this case.

## 2. Case Presentation

The patient was a 71-year-old woman who had been diagnosed as having slowly progressive type 1 diabetes in X-5 and systemic sclerosis associated with interstitial pneumonia in X-4. In October X-1, she was diagnosed as having rheumatoid arthritis. Although biological agents were introduced in addition to methotrexate (MTX), she had a secondary failure to respond to treatment with tocilizumab (TCZ) and a primary failure to respond to treatment with adalimumab (ADA) and had a decrease in activities of daily living owing to polyarthritis. In October, she was referred to our hospital and immediately admitted.

On admission, the disease duration of rheumatoid arthritis was 13 months. The patient presented with high disease activity and functional impairment as indicated by the following: tender joint (TJ) count, 21 (33); swollen joint (SJ) count, 20 (32); patient's global disease activity (PGA), 8.0 cm; evaluator's global disease activity, 8.2 cm; Clinical Disease Activity Index (CDAI), 57.2; Simple Disease Activity Index (SDAI), 68.8; disease activity score 28 based on C-reactive protein (CRP) level (DAS28-CRP), 7.61; and Health Assessment Questionnaire (HAQ) score, 2.00. Skin sclerosis in systemic sclerosis was present in the fingers and dorsal surface of the foot, and the modified Rodnan total skin thickness score (m-Rodnan TSS) was 8, which remained the same in December X-1. Her blood test results revealed higher levels of inflammatory markers and disease activity as follows: CRP level, 11.6 mg/dL; erythrocyte sedimentation rate (ESR), 104 mm/h; matrix metalloproteinase-3 (MMP-3) level, 715.3 ng/mL; serum amyloid A (SAA) level, 789 *μ*g/mL; rheumatoid factor (RF) level, 65 U/mL; and anti-cyclic citrullinated peptide (CCP) antibody level, 3472 U/mL. Finger radiography revealed joint space narrowing and bone erosions in both hands. Joint ultrasonography revealed multiple active synovitis. Chest computed tomography (CT) findings were consistent with interstitial pneumonia immediately below the pleura of the bilateral lower lobes; however, no increases in the levels of serum markers (KL-6 level, 320 U/mL; SP-D level, 24.4 ng/mL) were observed. Her diabetes was treated with insulin 4-2-2 units and insulin degludec 3-3-3 units at the time of admission. The test results were as follows: fasting plasma glucose level, 98 mg/dL; serum C-peptide level, 0.9 ng/mL; hemoglobin A1c (HbA1c) level, 7.4%; plasma C-peptide immunoreactivity (CPR) index, 0.9; pooled urine C-peptide level, 22.8 *μ*g/day; and anti-glutamic acid decarboxylase (GAD) antibody level, 1107 U/mL.

The diagnosis of rheumatoid arthritis was made in accordance with the 2010 American College of Rheumatology (ACR)/EULAR classification criteria as follows: swollen/tender joint count >10 joints (5 points), strongly positive anti-CCP antibody (3 points), disease duration >6 weeks (1 point), and high CRP/erythrocyte sedimentation (1 point), with a total score of 10 points. The diagnosis of systemic sclerosis was made in accordance with the 2013 ACR/EULAR classification criteria as follows: skin sclerosis of the fingers (skin sclerosis from the proximal interphalangeal joint to the metacarpophalangeal joint) (4 points), interstitial lung disease (2 points), and Raynaud's phenomenon (3 points), with a total score of 9 points (reference: fibrosis was present on skin biopsy of a subcutaneous tissue specimen). Computed tomography (CT) revealed mild interstitial pneumonia, but no pulmonary hypertension was found (estimated sPAP by cardiac ultrasonography, 32.7 mmHg). The diagnosis of slowly progressive type 1 diabetes was made in accordance with the diagnostic criteria of the Ministry of Health, Labour and Welfare (2012). The patient was anti-GAD antibody-positive and had a history of treatment with oral hypoglycemic drugs.

**Figure 1:**
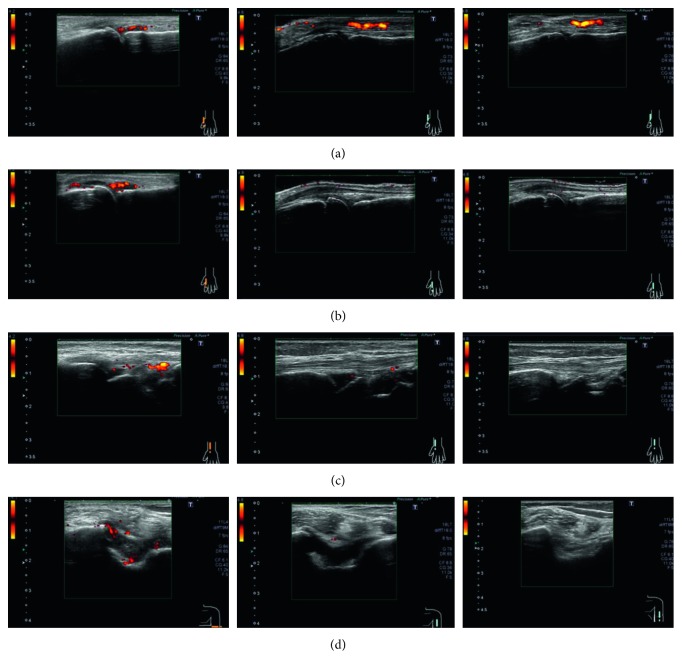
Joint ultrasonography findings. Left, before baricitinib administration; center, 24 weeks after the start of baricitinib administration; right, 52 weeks after the start of baricitinib administration. The abnormal findings in the following joints disappeared for up to 52 weeks in both PD and GS examinations: (a) left first MCP joint, (b) left second MCP joint, (c) left radial joint, and (d) left elbow joint (extensor surface/cross-sectional view). GS: grayscale; PD: power Doppler; MCP: metacarpophalangeal.

**Figure 2:**
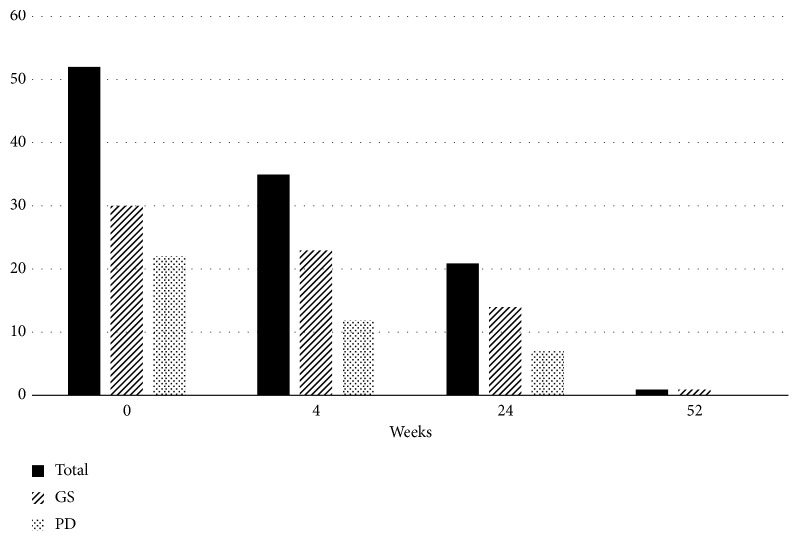
Changes in scores on joint ultrasonography (44 joints). A total of 44 joints were assessed with ultrasonography. The joint ultrasonography image shows significant improvement with time by baricitinib administration in the GS and PD examinations. Before introducing baricitinib: GS, 30 points, and PD, 23 points, with a total of 53 points. At 4 weeks after the introduction of baricitinib: GS, 23 points, and PD, 12 points, with a total of 35 points. At 24 weeks after the introduction of baricitinib: GS, 14 points, and PD, 7 points, with a total of 21 points. At 52 weeks after the introduction of baricitinib: GS, 1 point, and PD, 0 points, with a total of 1 point. Evaluation of the 44 joints included the DAS44 index (both sides of the hands, finger MCP joint, and IP/PIP, elbow, shoulder, sternoclavicular, acromioclavicular, knee, foot, and toe MTP joints). The severity grading of each joint in the GS and PD examinations was based on the classification by Szkudlarek et al. [8]. The total scores for each joint were calculated for GS and PD. GS: grayscale; PD: power Doppler; MCP: metacarpophalangeal; MTP: metatarsophalangeal; DAS44: disease activity score 44; IP: interphalangeal; PIP: proximal interphalangeal.

Treatment was switched to baricitinib, a JAK inhibitor, with concurrent use of oral MTX 12 mg/week. Her disease activity decreased rapidly 2 weeks after the start of treatment with baricitinib 4 mg/day; a steroid was concurrently used for 3 weeks (maximum prednisolone dose, 5 mg/day). The levels of the serological markers markedly improved with time as follows: anti-citrullinated protein antibody level markedly decreased; CRP level/ESR, RF level, MMP-3 level, and SAA level were normalized (Table 1). In addition, improvements in the blood flow signal and synovial thickening were observed on joint ultrasonography 4 weeks after the introduction of baricitinib, and further improvements were observed at 24 and 52 weeks (Figures 1 and 2). The skin sclerosis in systemic sclerosis was also improved. The m-Rodman TSS improved from 8 points to 2 points 24 weeks after the start of treatment, and no progression of skin sclerosis was observed at 52 weeks. Furthermore, high-resolution CT revealed that the progression of her interstitial pneumonia was controlled during the 52-week observation period, and the respiratory symptoms or dysfunction showed no progression. Regarding the influence on slowly progressive type 1 diabetes, the required daily dose of insulin decreased rapidly after the start of treatment (17⟶11 units), and the HbA1c level also decreased (7.4%⟶6.4%). The required insulin dose did not increase for up to 52 weeks, and the insulin secretory ability was almost unchanged (pooled urine CPR level, 22.8⟶21.8 *μ*g/day).

## 3. Discussion

Many inflammatory cytokines involved in rheumatoid arthritis mediate their effects via the JAK/signal transducer and activator of transcription (STAT) signaling pathways. The JAK family consists of JAK1, JAK2, JAK3, and TYK2, which are specifically associated with different types of cytokine receptors, among which baricitinib selectively inhibits JAK1/2 activity [9]. Baricitinib acts on interleukin 6 (IL-6), IFN-*γ*, granulocyte-macrophage colony-stimulating factor, and so on and suppresses the production of inflammatory cytokines [10]. To date, four international phase III trials of baricitinib have been conducted, reporting that the drug was highly effective for MTX-naive, MTX (or conventional synthetic DMARD)-resistant, and biologic DMARD-resistant patients with rheumatoid arthritis [11–14] and for suppressing the progression of joint destruction and functional impairment [11, 12]; however, no studies have reported the effectiveness on systemic sclerosis or type 1 diabetes in patients with rheumatoid arthritis.

The characteristics of the effectiveness of baricitinib in the present case are threefold, including suppression of disease activity of rheumatoid arthritis resistant to treatment with tumor necrosis factor (TNF)/IL-6 inhibitors, significant improvement in serological and imaging findings, and improvements in the skin sclerosis in systemic sclerosis and type 1 diabetes (decreases in the required insulin dose and HbA1c level).

The RA-BEAM trial, an international phase III clinical trial, demonstrated the effectiveness of baricitinib from the early stage of treatment in patients with rheumatoid arthritis insufficiently treated with MTX and not treated with biological agents [11, 15]. A subanalysis of the RA-BEACON trial reported the effectiveness of the drug in patients refractory to anti-TNF/anti-IL-6 agents [16]. The patient described herein did not respond to TNF/IL-6 inhibitors, but her disease activity was controlled by baricitinib in combination with a sufficient dose of MTX from the early stage of treatment. The SAA level was normalized by the treatment, although it had not decreased by the prior treatment with TCZ. This is considered to be caused by baricitinib, a JAK1/2 inhibitor, suppressing the phosphorylation of JAK2/STAT3 activated by IL-6 and thus suppressing the expression of SAA mRNA [17].

Regarding its influence on the skin sclerosis in systemic sclerosis, baricitinib, a JAK1/JAK2 inhibitor, was suggested to suppress the collagen production from fibroblast cells mediated by IL-6, among others [18, 19], and the activation of fibroblast cells mediated by TGF-*β* [10, 20, 21], which leads to improvement in the skin sclerosis.

Regarding the effects of JAK1/2 inhibitor on type 1 diabetes, a study using a mouse model of type 1 diabetes by Trivedi et al. reported that JAK1/2 inhibited the upregulation of major histocompatibility complex (MHC) class I expression in pancreatic *β*-cells mediated by IFN-*γ* and exerted a hypoglycemic effect from the early stage [22]. In the present case, the required insulin dose and HbA1c level decreased after baricitinib administration, and the patient's insulin secretion ability was maintained over a year, which suggests a possibility that the drug suppressed the destruction of pancreatic *β*-cells by CD8 + T cells via MHC class I.

In conclusion, the present case shows that baricitinib was effective for treating rheumatoid arthritis complicated by systemic sclerosis and type 1 diabetes. The improvements were observed in complicating diseases, namely, systemic sclerosis and type 1 diabetes. Baricitinib could be effective not only for rheumatoid arthritis but also for systemic sclerosis and type 1 diabetes. Further studies with accumulation of cases are needed.

## Figures and Tables

**Table 1:** Changes in rheumatoid arthritis disease activity indexes and levels of serological markers after the introduction of baricitinib.

Week	0	4	24	52
CDAI	57.2	4.0	6.0	6.5
SDAI	68.8	5.1	6.0	6.5
DAS28-CRP	7.61	3.00	2.29	2.34
CRP (mg/dL)	11.6	1.1	0.0	0.0
ESR (mm/h)	104	54	20	14
RF (IU/mL)	65	57	40	9
MMP-3 (ng/mL)	715.3	253.7	68.7	18.1
SAA (*μ*g/mL)	789.0	89.7	7.4	5.0
ACPA (IU/mL)	3472	—	—	329.1
HAQ	2.000	0.500	1.000	1.375

CDAI: Clinical Disease Activity Index; SDAI: Simple Disease Activity Index; DAS28-CRP: disease activity score 28 based on C-reactive protein (CRP) level; ESR: erythrocyte sedimentation rate; RF: rheumatoid factor; MMP-3: matrix metalloproteinase-3; SAA: serum amyloid A; ACPA: anti-citrullinated protein antibody; HAQ: Health Assessment Questionnaire.
